# Sorption Isotherm of Southern Yellow Pine—High Density Polyethylene Composites

**DOI:** 10.3390/ma8010368

**Published:** 2015-01-20

**Authors:** Feihong Liu, Guangping Han, Wanli Cheng, Qinglin Wu

**Affiliations:** 1 Key Laboratory of Bio-based Material Science and Technology (Ministry of Education), Northeast Forestry University, Harbin 150040, China; E-Mails: feihongliu8@gmail.com (F.L.); nefucwl@nefu.edu.cn (W.C.); 2 School of Renewable Natural Resources, Louisiana State University Agricultural Center, Baton Rouge, LA 70803, USA; E-Mail: wuqing@lsu.edu

**Keywords:** wood-plastic composites, sorption isotherm, durability treatments, nelson’s model

## Abstract

Temperature and relative humidity (RH) are two major external factors, which affect equilibrium moisture content (EMC) of wood-plastic composites (WPCs). In this study, the effect of different durability treatments on sorption and desorption isotherms of southern yellow pine (SYP)-high density polyethylene (HDPE) composites was investigated. All samples were equilibriumed at 20 °C and various RHs including 16%, 33%, 45%, 66%, 75%, 85%, 93%, and100%. EMCs obtained from desorption and absorption for different WPC samples were compared with Nelson’s sorption isotherm model predictions using the same temperature and humidity conditions. The results indicated that the amount of moisture absorbed increased with the increases in RH at 20 °C. All samples showed sorption hysteresis at a fixed RH. Small difference between EMC data of WPC samples containing different amount of ultraviolet (UV) stabilizers were observed. Similar results were observed among the samples containing different amount of zinc borate (ZB). The experimental data of EMCs at various RHs fit to the Nelson’s sorption isotherm model well. The Nelson’s model can be used to predicate EMCs of WPCs under different RH environmental conditions.

## 1. Introduction

Wood-plastic composites (WPCs) are extensively used for decking, railing, and fencing [1]. When WPCs are used outdoors, they are subjected to a climate with moisture and temperature-fluctuations, ultraviolet (UV) radiation, and decay fungi. After exposure of WPCs to varying environmental conditions, especially UV radiation from sunlight, the composites will undergo photo-degradation leading to undesirable effects, including a loss in mechanical properties and surface quality, *i.e.*, surface micro-cracking and color change [2,3,4]. The presence of moisture absorbed also aggravates this problem when WPCs are used for outdoor applications [5,6,7]. The existence of moisture in wood accelerates oxidation reaction, which is a direct consequence of photo-degradation [6]. The adverse effects of UV radiation on WPCs can be delayed or minimized with the use of UV stabilizers such as UV absorbers (UVA) and hindered amine light stabilizers (HALS) (free-radical scavengers). It was reported that the materials with UV stabilizers lightened less and showed less loss in mechanical properties compared with the materials without UV stabilizers [2]. Other studies showed that addition of HALS or UVA or both photo-stabilizers delayed and minimized the impacts of natural weathering on HDPE-wood fiber (WF) composites [8]. The stabilized HDPE-WF composite with UVA showed the best protection in the loss of flexural modulus among the test samples [8].

Another concern about the use of WPCs for outdoor applications is their fungal resistance. When the first generation of WPCs was applied into the market, it was considered to be immune to biological attack, because the fiber were encapsulated by plastic totally, and WPCs were water resistance. Actually, when WPCs are exposed in a humid atmosphere, its plastic-rich surface phase will delay the rate of moisture absorption; therefore the WPCs seem to be moisture resistance. However, it was reported that after a long-term exposure to high relative humidity (RH) surrounding, the surface layer of WPCs showed higher moisture content and the moisture distributed uniformly through the cross section of WPCs [9]. The high moisture content will provide an indispensable element for the growth of fungi and other microorganisms. Craig M. Clemons have studied the effects of processing method and moisture history on laboratory fungal resistance of wood-HDPE composites [10]. The results showed that significant weight losses were found once the composite moisture content reached roughly 12% to 15%, indicating severe decay [10]. Fungal degradation not only affected the aesthetic appearance of WPCs, but also decreased the mechanical properties and further affected the long-term performances of WPCs. Literatures showed that the inclusion of Zinc borate (ZB) can effectively prevent fungal decay and ZB will take at least 20 years to completely dissolve and leach from the material [11]. 

To date, many researches about the influence of adding ZB or UVA or HALS on the mechanical and weathering properties have been reported. However, the effects of adding ZB or UVA or HALS on the equilibrium moisture content (EMC) of WPCs under different RHs at a given temperature have not been reported yet.

Similar to solid wood, the wood component in WPCs absorbs and desorbs water molecules to be in equilibrium with the surrounding atmosphere, thus the EMC of WPCs varies with the change of the atmosphere. Mainly, the RH and temperature of the environment can affect EMC. The relationships between EMC and RH at given temperatures, *i.e.*, sorption isotherms, greatly affect the performances of wood-based materials including strength and dimensional stability in their applications. Many studies have been done with the moisture sorption isotherm and the models to evaluate the EMC of solid wood [12,13,14,15,16]. However, reports on EMC of WPCs under different environments are limited, especially the isotherm models of EMC as a function of temperature and RH. Only Adebola studied the absorption and desorption performances of two commercial WPCs [17]. In his study, the experimental EMC data were compared with Hailwood-Horrobin (H-H) sorption model predictions using the same temperature and RH. The results showed that the amount of moisture absorbed increased with the increase of RH at an average temperature of 23 °C, and the predicted EMC values of WPCs deviated from the H-H model. The H-H model was shown to be ineffective in predicting the EMC of WPCs [17]. 

One of the most common used models to describe sorption isotherms is Nelson’s model based on Gibbs free energy [18]. Wu applied this model to medium density fiberboards and oriented strand boards, and the results showed that it could be used to describe the sorption property of wood-based boards [19]. The similar results indicated that it also could be applied to straw particleboards [20]. Han *et al.* [21] compared the sorption property of sugarcane rind with that of wood strand for structural use. It demonstrated that the model could be used as a tool for predicting moisture change in wood-based products under varying environmental conditions. However, the applicability of Nelson’s model to predict the sorption isotherm of WPCs is not clear yet.

Accordingly, the primary objectives of this study were to investigate: (a) the sorption and desorption properties of WPCs; (b) the effects of different durability treatments, *i.e.*, the addition of ZB and UV stabilizers, on the EMC of WPCs under various RHs; and (c) the effectiveness of Nelson’s model in predicting EMC of WPCs. In addition, sorption isotherms reproduced by Nelson’s model of WPCs were compared with other composite materials and solid wood.

## 2. Experimental

### 2.1. Raw Materials and Preparation

High-density polyethylene (HDPE) (HD0760, density = 960 kg/m^3^, melt flow index (MFI) = 0.7 g/10 min at 190 °C/2.16 kg) from ExxonMobile Chemical Co. (Houston, TX, USA) was used as a matrix. Southern yellow pine (SYP) wood flour with a particle size of 20 mesh was provided by American Wood Fiber Inc. (Schofield, WI, USA). Maleated polyethylene (MAPE) (EpoleneTM G2608 with molecular weight = 65,000 g/mol, MFI = 6 to 10 g/10 min at 190 °C/2.16 kg) was applied to increase the compatibility between wood fibers and the plastic matrix. Zinc borate (ZB) as a preservative was purchased from the US Borax Co. (Greenwood Village, CO, USA). Lubricant (TPW 306) from Struktol Co. (Stow, OH, USA) was used for easy processing of the WPC profile. UVA (Tinuvin 326) and HALS (Tinuvin 783 and Chimassorb 944), supplied by Ciba Specialty Chemicals Inc. (Mississauga, ON, Canada), were chosen for this study. The UV absorber (Tinuvin 326) acts by absorbing UV radiation preferentially to polymers; and the two HALS classes (Tinuvin 783 and Chimassorb 944) are based on antioxidants. Colorant was also added to provide the composites with a wood-like appearance. All the chemical additives were used as received.

### 2.2. Blend Design and Sample Fabrication

The composite blends were prepared using a Leistritz Micro-27 co-rotating parallel twin-screw extruder (Leistritz Corporation, Allendale, NJ, USA). The extrusion temperatures were controlled at 155, 160, 165, 170, 170, 170, 160, 150, 150, 150, and 155 °C from the feeing zone to die. The extruder rotation speed was set at 60 rpm. HDPE, wood flour, UV stabilizers, ZB, and other processing aids (MAPE 2%, Talc 5%, Lubricant 5%, Colorant 2%) were added to the extruder and thoroughly mixed. Profile extrusion was conducted using an Intelli-Torque Twin-Screw Extruder (CW Brabender Instruments; South Hanckensack, NJ, USA) and a die (5 mm × 50 mm). The temperatures for the profile extrusion was from 150 (feeder), 165, 160, and 155 °C (die). The profile was cut into various lengths for further testing after air cooling. Table 1 shows the design for various blends. 

**Table 1:** Wood flour and high density polyethylene (HDPE) blend design for the wood-plastic composite (WPC) samples.

Material type	UV stabilizer (wt%)			ZB (wt%)	Wood flour (wt%)	HDPE (wt%)	Other processing aids (wt%)
	Tin783	Chi944	Tin326				
1	–	–	–	–	55	31	14
2	1	–	–	–	55	30	14
3	2	–	–	–	55	29	14
4	–	1	–	–	55	30	14
5	–	2	–	–	55	29	14
6	–	–	1	–	55	30	14
7	–	–	2	–	55	29	14
8	1	–	–	1	55	29	14
9	2	–	–	2	55	27	14
10	–	1	–	1	55	29	14
11	–	2	–	2	55	27	14
12	–	–	1	1	55	29	14
13	–	–	2	2	55	27	14
14	–	–		1	55	30	14
15	–	–		2	55	29	14

### 2.3. EMC Measurements

Four specimens from each of the material types were randomly selected and numbered and then combined to form one group. The size of all samples is 10 mm × 10 mm × 4 mm. For absorption, eight groups of samples were randomly selected and oven dried at 70 °C for two days to reach the dry state. The remaining eight groups were conditioned over distilled water to reach the fiber saturation state for the desorption experiment. All groups of samples were conditioned at a relative humidity of 16%, 33%, 45%, 66%, 75%, 85%, 93%, and 100% respectively. The RHs of 16% and 45% were achieved over sulfuric acid solutions with concentrations of 60% and 45%, respectively. While the RH of 100% was generated through distilled water placed in desiccators when it reached the equilibrium state. Five desiccators charged with saturated salt solutions were used to obtain other five specified conditions. The weights of all specimens were measured after 4 months when the specimen reached equilibrium. After conditioning, the specimens were oven dried at 103 °C for 24 h. The EMC of each specimen was calculated based on the oven dry weight using Equation (1).

(1) $$EMC=\left(\frac{w_{t}}{w_{o}}-1\right)\times 100\%$$

where *w_t_* is the equilibrium weight of sample (g) and *w_o_* is the oven dry weight of sample (g).

### 2.4. Nelson’s Model

Experimental data of EMC at various RHs were fit to the sorption isotherm model proposed by Nelson (1983). The model is of the form:

(2) $$\frac{RH}{100}=exp\;\left\{\left(-\frac{W_{w}}{RT}\right)exp\left[A\left(1.0-\frac{EMC}{M_{v}}\right)\right]\right\}$$

where: *RH* = Relative humidity in percent; exp = Exponential function; *M_v_* = Material constant which approximates the fiber saturation point for desorption (%); *A* = Natural logarithm of the Gibbs free energy per gram of sorbed water as RH approacheszero (∆G_o_, cal/g), *i.e.*, A = ln(∆G_o_); *R* = Universal gas constant (1.9858 cal/mole/°K); *T* = Absolute temperature (°K); *W_w_* = Molecular weight of water (18.1 g/mol). At a given temperature, the term (−*RT*/*W_w_*) becomes a constant, and then parameters *A* and *M_v_* define the sorption isotherm. To determine the values of *A* and *M_v_*, a linear regression analysis was performed using Equation (3) with the measured EMC as the dependent variable and transformed RH, *i.e.*, *Ln[(–RT/W_w_*) *Ln(RH*)] as the independent variable:

(3) $$EMC=M_{v}\left\{1.0-\frac{1}{A}Ln\left[\left(-\frac{RT}{W_{W}}\right)LnRH\right]\right\}$$

## 3. Results and Discussion

### 3.1. Sorption Isotherm of WPCs

**Figure 1:**
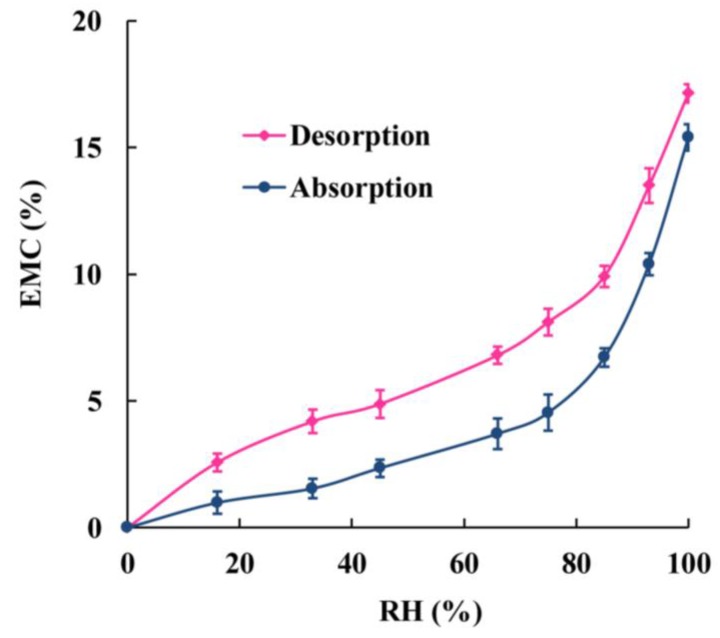
Typical sorption and desorption isotherms for type 1 WPC samples at 20 °C. Material types refer to Table 1.

Figure 1, based on the EMC data both from absorption and desorption, shows that EMC values increased with increasing RH for all samples both from absorption and desorption. Notably, the EMC data achieved through the absorption process were lower than those obtained from desorption at the same RH, indicating sorption hysteresis. This result was similar to the previous studies on wood-based materials including oriented straw board (OSB), middle density fiberboard (MDF), and solid wood [18]. Compared to solid wood, WPCs are kind of more complex composites with a combination of wood fibers and thermoplastic polymers. For WPCs, wood fibers are partly encased by the plastic, so the pathway, which water molecules come through, is changed accordingly. For solid wood, water molecules diffuse through wood cell wall, capillary tubes, and micro-capillary tubes [1,2]. The routes of water molecules diffusion into WPCs are the gaps and the interfaces between wood fibers and plastic matrix resulted from poor interface compatibility and the micro-cracks formed in the process of compounding first. And then water molecules transmission was similar to solid wood when the moisture was captured by the wood fibers in the WPCs. Although the channels of moisture molecules coming into the WPCs and solid wood are different, water molecules are absorbed by the wood fibers finally. Therefore, the WPCs samples showed similar hysteresis phenomenon with solid wood resulting from the wood fibers in WPCs.

### 3.2. Effects of Durability Treatment on the EMC of WPCs

**Figure 2:**
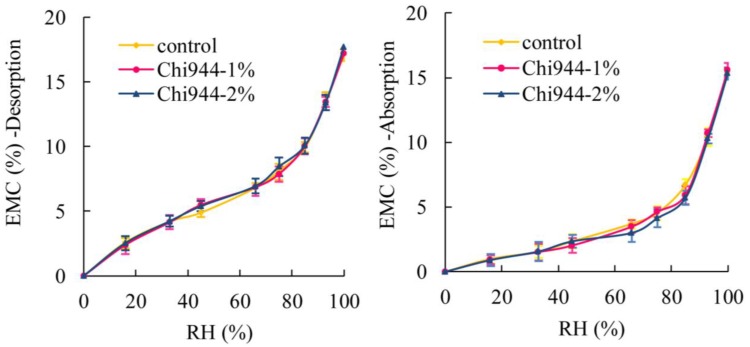
Effects of UV stabilizers Chi944 on equilibrium moisture content (EMC) of WPCs.

**Figure 3:**
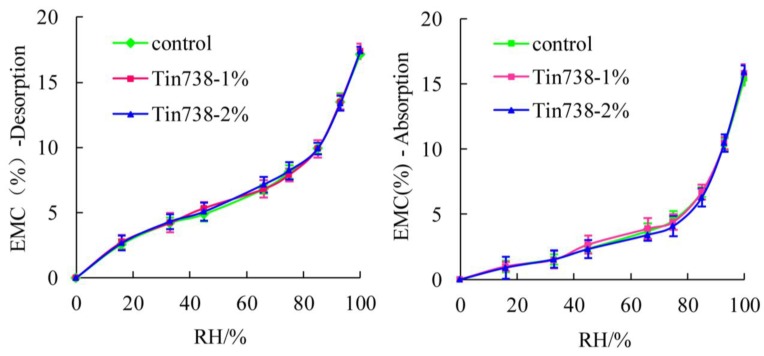
Effects of UV stabilizers Tin738 on EMC of WPCs.

**Figure 4:**
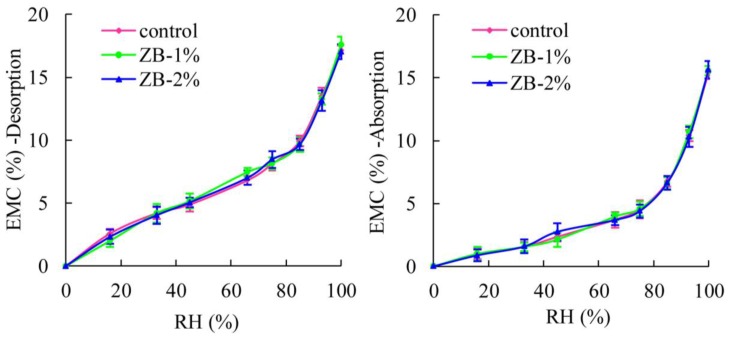
Effects of zinc borate (ZB) on EMC of WPCs.

Figure 2 shows the EMC values achieved from absorption and desorption of WPC samples that contain Chi944 (HALS) UV-stabilizers. EMC values of WPCs were increased with the increase of RH for all samples both from desorption and absorption. For desorption, regardless of the amount of UV-stabilizers added, WPCs samples treated by Chi944 had approximately the same EMC data as the control samples (samples without any durability treatments) at a given RH. Similar case was observed for absorption. The effects of UV stabilizers Tin738 (UVA) on the EMC values of WPCs samples (Figure 3) were similar to that of Chi944. These two Figures suggest that effects of UV-stabilizers on the EMC values of WPCs were slight. Figure 4 shows that the EMC values of WPCs were not affected by the addition of ZB for both absorption and desorption.

Different durability treatments had only slight effects on the EMC of the composites. The moisture uptake is almost a constant when the amount of wood fibers in WPCs is fixed. In this study, the wood content was 55% by weight for all material types while the maximum content of ZB or UV stabilizers was only 2%, which is so small that the effects of them on the moisture sorption property of WPCs could be ignored. In other words, the sorption isotherm of WPCs in the study was dominated by wood fibers.

### 3.3. Nelson’s Model Fit to Measured EMC

**Figure 5:**
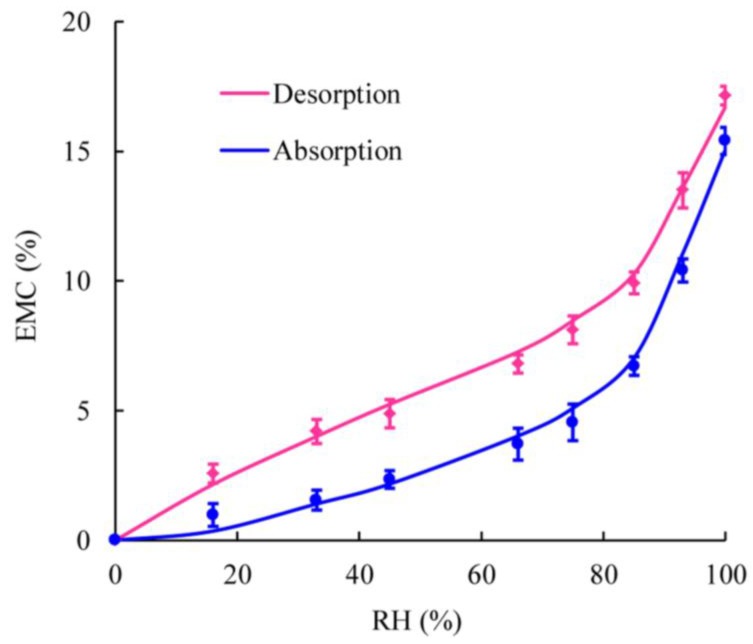
Comparison between calculated EMC by Nelson’s model and measured EMC from experiments. Lines show values predicated by the model.

Figure 5 shows typical graphs of the comparison between measured and predicted EMC data for WPCs (based on the data of WPC samples for type 1). Lines show sorption and desorption isotherm predicated by the model. The experimental EMC data were close to that of predicted by Nelson’s model, presenting agreement with the model. Apparently, the absorption curve was lower than the desorption curve, indicating sorption hysteresis at a given RH level. 

**Figure 6:**
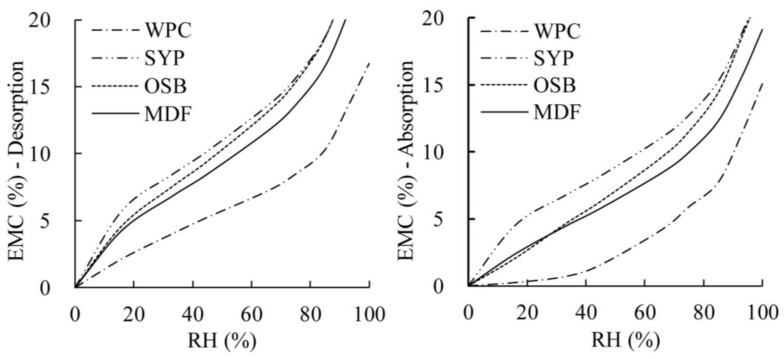
Comparison of sorption isotherm predicated by Nelson’s model for different types of materials.

The regression analysis data on sorption isotherm for various materials is shown in Table 2. The values of parameters *A* and *Mv* were different for various materials. These parameters can be used in Equation (3) to predict the EMC of different materials at a given RH level. The magnitude of *Mv* was higher in desorption than in absorption for all materials tested at a given RH. Among all these materials, WPCs showed the lowest magnitude of *Mv* both in absorption and desorption, averaging at 16.70 for absorption and 16.81 for desorption. Composite panels and solid wood had higher *Mv* values than WPCs. Figure 6 shows the comparison of sorption isotherm predicated by Nelson’s model based on the parameters listed in Table 2 for different material types from desorption and adsorption. The sorption isotherm of WPCs is lower than that of the other three material types both for adsorption and desorption. It is demonstrated that WPCs is less accessible to water than solid wood and wood composite products under the same environment. This difference may be related to the characteristic of the materials. Compared to solid wood and wood composites, the wood components in WPCs are partial covered by the water-resistant plastic, which prevent the wood fibers from absorbing the water. In addition, WPCs contain only 55% of wood fiber by weight while the moisture content is calculated by the total weight of the WPCs, thus the EMC of WPCs are relatively smaller than solid wood and wood composites.

**Table 2:** Parameters of Nelson’s sorption isotherm for different materials.

Material	Absorption		Desorption		Hysteresis ratio (Ads./Des.)
	*A* (cal/g)	*M_v_* (%)	*A* (cal/g)	*M_v_* (%)	
WPC	3.37	16.70	4.60	16.81	0.978
MDF ^a^	4.68	19.13	4.94	24.94	0.767
Aspen ^a^ OSB ^a^	4.49	22.94	4.89	28.28	0.8111
SYP ^a^	5.11	22.66	5.17	27.60	0.821

^a^ data are from Wu (1999) [19] at 25 °C.

## 4. Conclusions

Within the scope of this study, the main conclusion is that WPCs demonstrate sorption and sorption hysteresis phenomena similar to wood and other wood-based composites. The other conclusions can also be drawn as follows:

- The additives such as ZB and UV stabilizers at the loading levels used have little effect on the EMC of WPCs under various RHs at 20 °C;
- The experimental data of EMC at various RHs fit to Nelson’s sorption isotherm model well and the model can be used to predict EMCs of WPCs under different RH environmental conditions;
- The EMC of WPCs is significantly lower than solid wood and other wood-based composites due to less wood content and partially encapsulation of the wood fiber by plastic in WPC.
